# Machine learning classification of mediastinal lymph node metastasis in NSCLC: a multicentre study in a Western European patient population

**DOI:** 10.1186/s40658-022-00494-8

**Published:** 2022-09-24

**Authors:** Sara S. A. Laros, Dennis Dieckens, Stephan P. Blazis, Johannes A. van der Heide

**Affiliations:** 1 Department of Medical Physics and Engineering, Albert Schweitzer Hospital, Afdeling Klinische Fysica - Medische Techniek, Albert Schweitzerplaats 25, 3318 AT Dordrecht, The Netherlands; 2 Department of Nuclear Medicine, Albert Schweitzer Hospital, Dordrecht, The Netherlands; 3 Department of Nuclear Medicine, Diakonessenhuis Hospital, Utrecht, The Netherlands; 4 Department of Nuclear Medicine, University Hospital RWTH, Aachen, Germany

**Keywords:** Lung cancer, Intrathoracic lymph nodes, Machine learning, Radiomics, PET-CT

## Abstract

**Background:**

[^18^F] FDG PET-CT has an important role in the initial staging of lung cancer; however, accurate differentiation between activity in malignant and benign intrathoracic lymph nodes on PET-CT scans can be challenging. The purpose of the current study was to investigate the effect of incorporating primary tumour data and clinical features to differentiate between [^18^F] FDG-avid malignant and benign intrathoracic lymph nodes.

**Methods:**

We retrospectively selected lung cancer patients who underwent PET-CT for initial staging in two centres in the Netherlands. The primary tumour and suspected lymph node metastases were annotated and cross-referenced with pathology results. Lymph nodes were classified as malignant or benign. From the image data, we extracted radiomic features and trained the classifier model using the extreme gradient boost (XGB) algorithm. Various scenarios were defined by selecting different combinations of data input and clinical features. Data from centre 1 were used for training and validation of the models using the XGB algorithm. To determine the performance of the model in a different hospital, the XGB model was tested using data from centre 2.

**Results:**

Adding primary tumour data resulted in a significant gain in the performance of the trained classifier model. Adding the clinical information about distant metastases did not lead to significant improvement. The performance of the model in the test set (centre 2) was slightly but statistically significantly lower than in the validation set (centre 1).

**Conclusions:**

Using the XGB algorithm potentially leads to an improved model for the classification of intrathoracic lymph nodes. The inclusion of primary tumour data improved the performance of the model, while additional knowledge of distant metastases did not. In patients in whom metastases are limited to lymph nodes in the thorax, this may reduce costly and invasive procedures such as endobronchial ultrasound or mediastinoscopy procedures.

## Background

Non-small cell lung cancer (NSCLC) is one of the most prevalent malignancies in the western world; PET-CT has an important role in its initial staging [1–5]. The use of [^18^F] FDG PET-CT for preoperative staging of NSCLC reduces both the total number of thoracotomies and the number of unnecessary thoracotomies [6–9]. It has high sensitivity and intermediate specificity for the detection of primary tumours, locoregional lymph node metastases, and distant metastases. The limited specificity of PET-CT for lymph node metastases necessitates pathological confirmation, mostly obtained through the use of endobronchial ultrasound or mediastinoscopy procedures [10–16], which are costly and carry a risk of complications.

Artificial intelligence has the potential to improve diagnostic accuracy and management in patients with NSCLC [17–20], and specifically for use with CT imaging [21–23]. Wang et al. [24] demonstrated that a classification model for PET-CT, based on machine learning (ML) methods, can be highly accurate and perform on par with or better than human observers in differentiating between malignant and benign intrathoracic lymph nodes. Yoo et al. [25] included primary tumour data and achieved better performance, using a boosted decision tree in a single-centre study in a Korean population.

The aim of this study was to use an improved gradient boost algorithm-based implementation of gradient boosted classification models for the supervised classification of intrathoracic lymph nodes in patients with NSCLC in a retrospective multicentre study in Dutch hospitals. We also investigated the effect of additional clinical record information of distant metastases to differentiate between [^18^F] FDG-avid malignant and benign intrathoracic lymph nodes. We only made use of open-source and readily available free software tools for the selection of radiomic features, training, and evaluation of our classification model.

## Methods

### Patient group

We included 148 consecutive patients, diagnosed with NSCLC, who underwent a clinically indicated PET-CT scan for primary staging between July 2017 and July 2019 in the Albert Schweitzer hospital in Dordrecht (centre 1) and between July 2014 and December 2019 in the Diakonessen hospital in Utrecht (centre 2). For patient characteristics, see Table 1. The decision to request a PET-CT for primary staging of NSCLC was made at the discretion of the referring pulmonologist. All patients were scanned 60 min after intravenous administration of 2–3 MBq/kg of [^18^F] FDG, after which a low-dose CT was performed for attenuation correction and anatomical reference. At both centres, the PET-CT acquisitions were scanned using a Biograph mCT PET-CT system, manufactured by Siemens Healthcare (Erlangen, Germany). The clinical acquisition protocol at both sites consisted of a scanning time of 3 min per bed position and a similar iterative reconstruction of the acquired data incorporating the point spread function and time-of-flight corrections.

**Table 1:** Patient characteristics

	Centre 1	Centre 2
Number of patients	118	30
Age (average ± SD)	69 ± 9 years	67 ± 11 years
Male/female	67/51	20/10
Blood glucose level (average ± SD)	5.8 ± 1.1 mmol/L	6.4 ± 2.2 mmol/L
*Smoking status* Current/ex/never/unknown	37/57/5/19	18/11/1/0
*Tumour type* Adeno/squamous/large cell/NOS/unknown	41/41/22/10/4	12/4/4/4/6
Stage: I/II/III/IV	12/20/48/37	3/2/12/13

Patients who had [^18^F] FDG uptake above background in intrathoracic lymph nodes underwent biopsy, mostly with an endobronchial ultrasound-guided fine-needle aspiration, within one week after PET-CT scan. A small group underwent surgical biopsy, either during the mediastinoscopy or at the definitive surgery.

### Image pre-processing

The acquired and reconstructed image volumes were anonymized and transferred to a workstation for further analysis. The primary tumour and suspected lymph node metastases were annotated using Osirix MD software (Pixmeo SARL, Geneva, Switzerland). PET images and fused PET-CT images were used for annotation. When two lymph nodes were fused at a border, the borders were adhered to separate the various lymph node stations, as described in the 2009 IASLC lymph node map [26]. The annotation was performed by a nuclear medicine physician with 8 years of experience in reading PET-CT scans. After cross-referencing with pathology results obtained with biopsy or surgery, lymph nodes were classified as malignant or benign. Lymph nodes without pathologic confirmation were excluded from the analysis, see Table 2. The resulting 504 samples of malignant and benign lymph nodes were combined with various clinical data from the corresponding patients and were subsequently used as the dataset for the creation and analysis of the classification model.

**Table 2:** Overview of included lymph nodes and primary tumours

	Centre 1	Centre 2	Total
Number of primary tumours	118	30	148
Malignant lymph nodes	312	75	387
Benign lymph nodes	94	23	117

The lymph nodes and primary tumours were extracted from the PET volumes by using the segmentation masks from the manual segmentation step mentioned above. All segmented volumes were padded with zeros to create volumes with the dimensions 144 × 144 × 144.

**Fig. 1:**
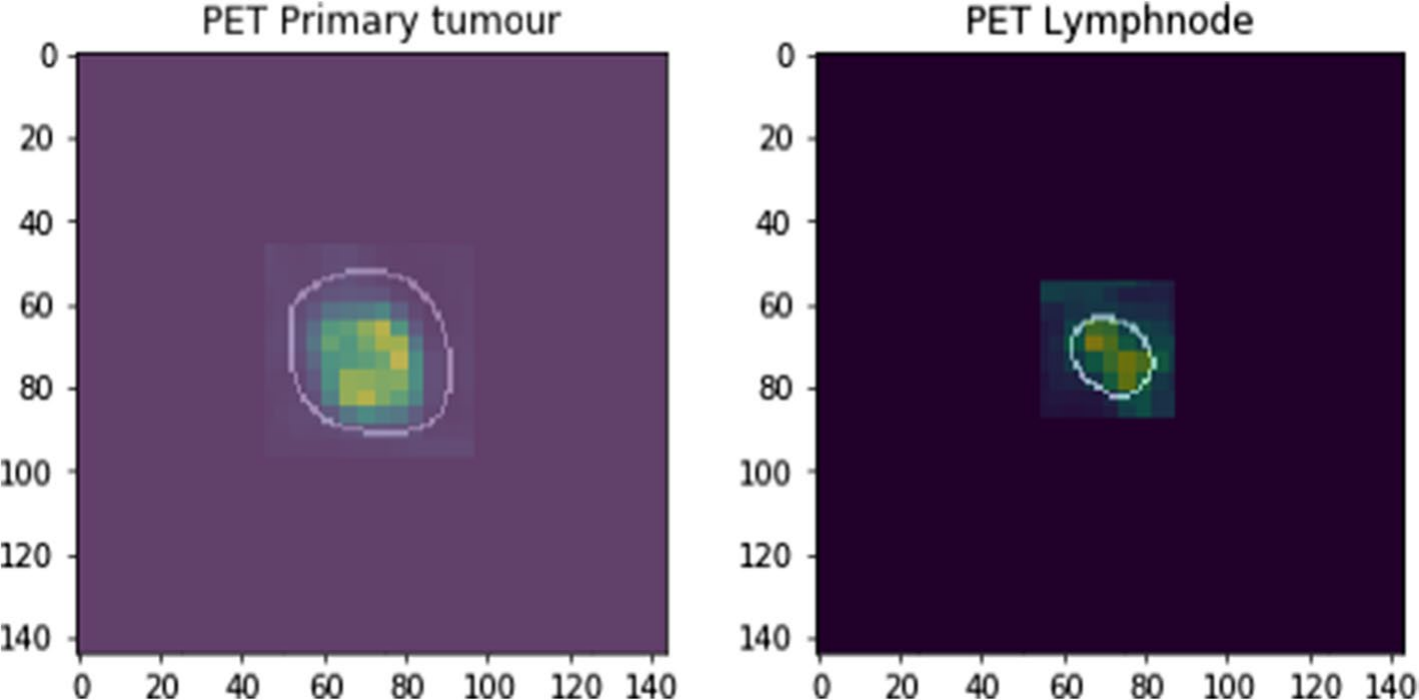
Example of the input data. Each input dataset consisted of a manually segmented primary tumour and a manually segmented lymph node. All data were padded with zeros to have the same size of 144 × 144 × 144 voxels

After normalization of the PET images, we created input data records for each lymph node of the dimensions 144 × 144 × 144. The input data record for each lymph node contained the lymph node PET image and the lymph node segmentation mask (as shown in Fig. 1), and we combined these images with the primary tumour PET image and the primary tumour segmentation mask.

### Radiomic features and machine learning methods

To establish a baseline, we first analysed the performance of a simple standardized uptake value (SUV) threshold model based on the mean SUV of the lymph nodes. Boosted decision trees have previously been shown to be very successful for the classification of intrathoracic lymph nodes [24, 25]. A specific implementation of the gradient boosting method is the extreme gradient boosting (XGB) algorithm. This algorithm has been shown to be even more successful [27], and therefore, we used the XGB algorithm.

For both the primary tumours and lymph nodes, we derived diagnostic features from the PET data using the PyRadiomics library (version 3.0.1). This open-source library extracts a standard set of features and, therefore, improves reproducibility [21]. The features can be subdivided into 7 different feature groups: first-order statistics, shape-based (3D), grey-level co-occurrence matrix, grey-level size zone matrix, grey-level run length matrix, neighbouring grey tone difference matrix, and grey-level dependence matrix features. In each feature group, a different number of features was available to be calculated. In total, 107 features were calculated for each of the two different data inputs (primary tumour PET and lymph node PET image data). All features were included.

To study the effects of the absence or inclusion of the primary tumour and clinical information into our models, we defined various scenarios by selecting different combinations of data input (primary tumour PET and lymph node PET) combined with different clinical features (all clinical features, only metastases features, and no clinical features), which resulted in six scenarios. The model in which all clinical features were included contained the patient’s age, sex, smoking status, and, in the case of metastases, specific information about the type of metastasis (abdominal, bone, adrenal, liver, or thyroid). In all scenarios, the data from centre 1 were used for training and validation of the models (see Table 1). To train and validate the models, we used a 10 times repeated stratified fivefold validator. This cross-validation method splits the data into five different subsets in which the training set consisted of four subsets and the validation set consisted of one subset. This process was repeated until the model was trained and validated by all different combinations of subsets. Besides that, we studied the impact of including less features in the model and, therefore, we created three additional scenarios that contained the 5, 10, and 20 best-performing features. To determine the best-performing features, the model was trained (10 times repeated stratified fivefold) and, then, the feature weights were calculated. The top 5, 10, and 20 best-performing features were selected as model features. Those models were compared to the model in which all features were included.

To compare various models, we determined the receiver operator characteristic (ROC) curves. The effect of the absence or inclusion of the primary tumour information and clinical information was evaluated by the area under the ROC curve (AUC). The AUCs were analysed with the independent two sample t test. The performance of the best model was evaluated for the validation set (10 times repeated stratified fivefold validator) by the diagnostic accuracy (ACC), the negative predictive value (NPV), positive predictive value (PPV), specificity (SPEC), and sensitivity (SENS). To determine the performance of the model in a different hospital, the model was tested using the data from centre 2 (see Table 1) and evaluated by the ACC, NPV, PPV, SPEC, and SENS. Python software and scikit-learn toolkit were conducted for establishment and evaluation of the models. A *p* value of less than 0.05 was considered significant.

## Results

### Distribution of SUV

**Fig. 2:**
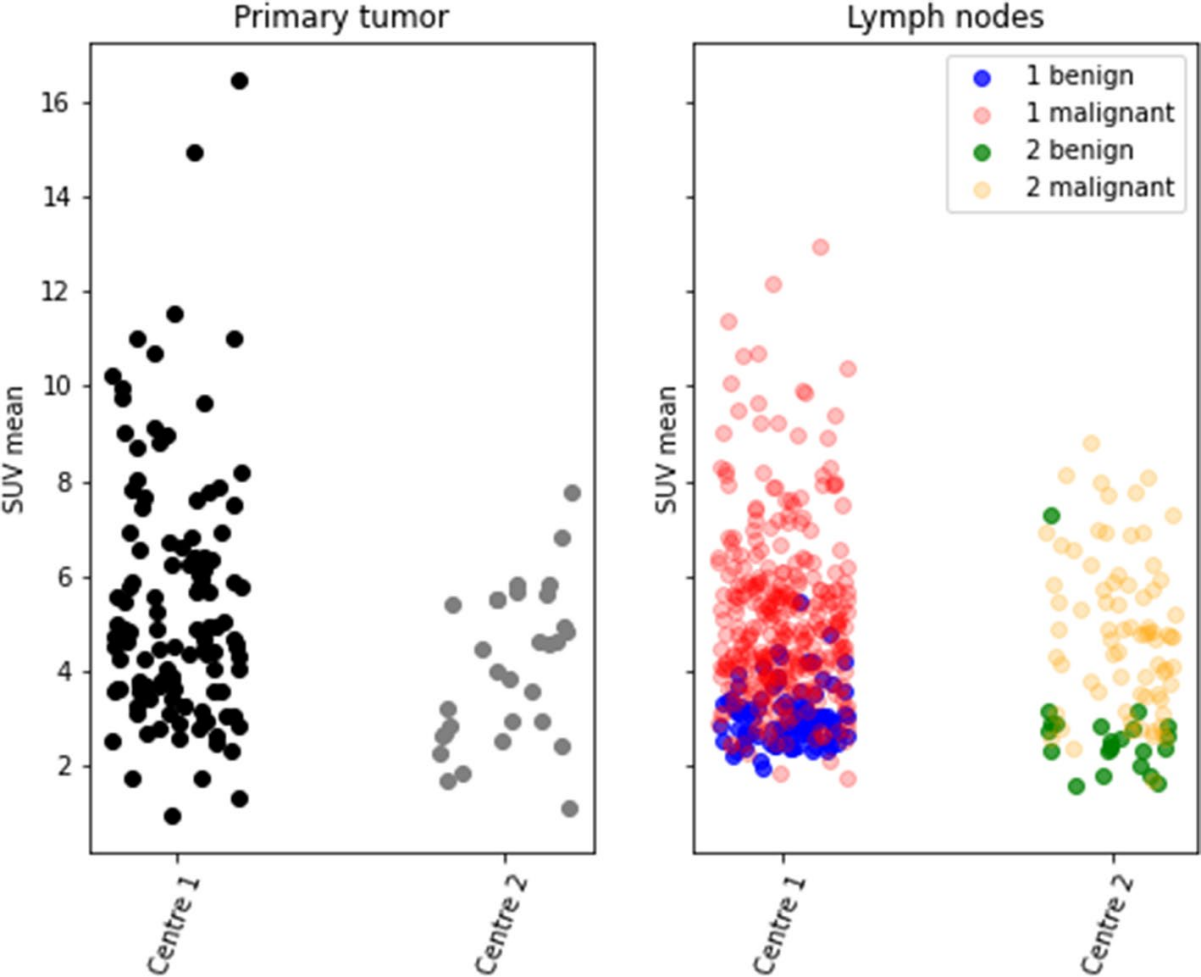
Overview of the SUV-mean of the datasets from both hospitals (ASZ and DK) used in this study. The left-hand side shows the distribution of SUV-mean of the primary tumours. The right-hand side shows the distribution of SUV-mean of benign and malignant lymph nodes

For each patient, we determined the SUV of both the primary tumour and the lymph nodes for which pathology results were present. As shown in Fig. 2, the distributions of the SUVs of the malign and benign groups of lymph nodes differed significantly.

### ROC curves

**Fig. 3:**
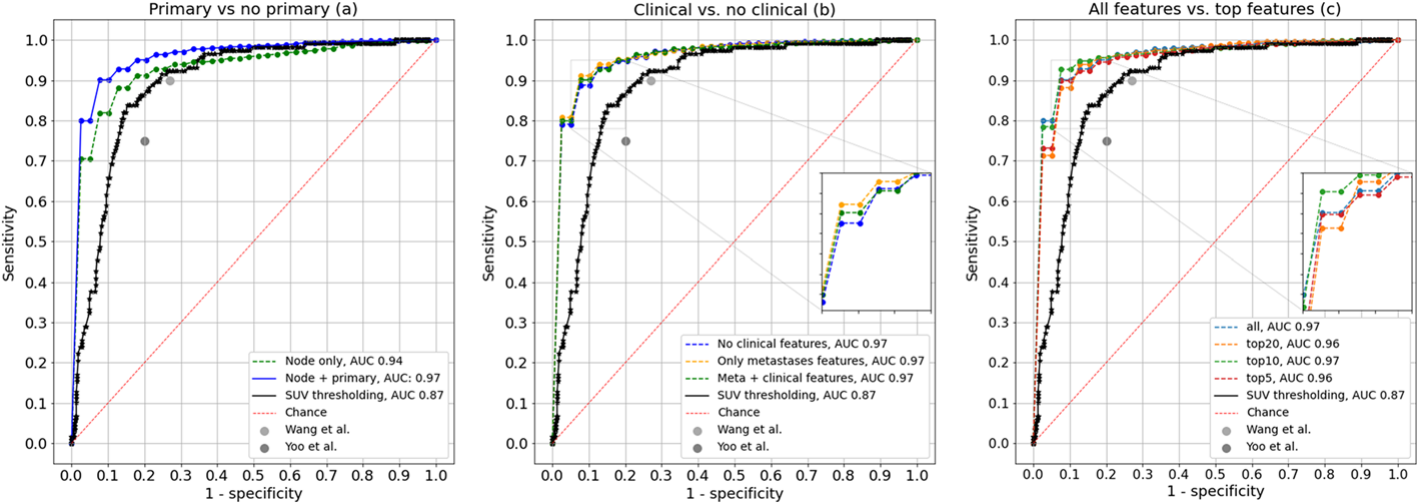
Overview of ROC curves for the XGB classifier models with and without the inclusion of primary tumour features (left), with and without the inclusion of additional clinical knowledge (middle) and a selection of features (right). For reference, the SUV threshold model is also shown. The performance of doctors is taken from Wang et al. [24] and Yoo et al. [25]

We determined the ROC curves and AUC values for the classifier models, which were trained and validated using data from centre 1. All the classifier models significantly outperformed the simple threshold model based on the mean SUV, see Fig. 3. For reference, we also included the results of the human observer studies as reported by Wang et al. [24] (sensitivity 90% and specificity 73%) and Yoo et al. [25] (sensitivity 75% and specificity 80%). The results showed that adding primary tumour information resulted in a significant gain in performance compared to using the node image data only (Fig. 3a). Adding the clinical information, including distant metastases, did not lead to significant differences in AUC values (Fig. 3b). No significant differences were observed between the model using all features or only the top 5, 10, and 20 best-performing features; however, the model including all features and only the top 10 best-performing features showed a small increase in AUC compared to the top 5 and top 20 models (Fig. 3c). To prevent overfitting, the model using the top 10 best-performing features was selected as the best-performing model. This model consisted of radiomic features using data input from PET lymph nodes as well as PET primary tumour data. An overview of the top 5, 10, and 20 best-performing features is shown in Table 3.

**Table 3:** Overview of the top 5, top 10, and top 20 best-performing features and the permutation feature importance

	Feature	Data input		Feature weight
		PET lymph node	PET primary	
1	90 Percentile	X		0.63
2	Sphericity		X	0.16
3	Elongation		X	0.08
4	Variance	X		0.07
5	Least axis length		X	0.06
6	Grey-level non-uniformity		X	0.06
7	Short-run high grey-level emphasis		X	0.03
8	Energy		X	0.02
9	Short-run emphasis	X		0.02
10	Dependence non-uniformity		X	0.02
11	Maximum 2D diameter row	X		0.02
12	Interquartile range	X		0.02
13	Grey-level non-uniformity normalized		X	0.01
14	Large dependence high grey-level emphasis		X	0.01
15	Short-run emphasis		X	0.01
16	Robust mean absolute deviation	X		0.01
17	Elongation	X		0.01
18	Minimum		X	0.01
19	Mean absolute deviation	X		0.01
20	Maximum		X	0.01

### Performance indicators

For the model using the top 10 best-performing features, the ACC, NPV, PPV, SPEC, and SENS have been summarized for the data in centre 1 (validation set) and centre 2 (test set), see Table 4. The performance of the model in the test set was slightly but statistically significantly worse than in the validation set.

**Table 4:** Performance results in centre 1 (mean ± st.dev.) and centre 2 of the XGB model using the top 10 best-performing features

	Centre 1 (validation set)	Centre 2 (test set)
ACC	0.92 ± 0.02	0.88
NPV	0.95 ± 0.03	0.90
PPV	0.83 ± 0.09	0.70
SENS	0.85 ± 0.07	0.80
SPEC	0.95 ± 0.03	0.90

## Discussion

In patients with NSCLC, [^18^F] FDG PET-CT has a high sensitivity but intermediate specificity in the detection of intrathoracic lymph node metastases. In clinical practice, this means that even in lymph nodes with very little [^18^F] FDG uptake, metastasis cannot be ruled out and pathologic confirmation is necessary. Pathologic confirmation usually requires mediastinoscopy or endobronchial ultrasound-guided fine-needle aspiration, which are costly and carry a risk of complications. The present study was conducted to find whether the XGB-based model could differentiate between malignant and benign lymph nodes in PET-CT in a clinical setting. We investigated the effect of the absence or inclusion of the primary tumour data, as well as clinical data into our models in a retrospective two-centre study in Dutch hospitals. The performance of the model was tested on a completely separate data set from a different hospital.

The inclusion of primary tumour data improved the performance of the model, but additional knowledge of distant metastases and other clinical data did not. Optimal performance was achieved using only the top 10 best-performing features. When the model was tested using data from centre 2, the mean NPV decreased from 0.95 to 0.90. As expected, using data from another hospital resulted in a small decrease in ACC, NPV, PPV, SPEC, and SENS. An explanation for this might be small differences in practical implementation between both hospitals, such as the temperature in the patient resting room, the distance between the resting room and the scanner room, and the exact time between the injection and the start of the scan. Furthermore, there were small differences in patient characteristics between centre 1 and centre 2. Centre 2 consisted of relatively more men, more smokers, more adenocarcinomas, and a higher blood glucose level. Although those differences in practical implementation and patient characteristics will not lead to big differences in image quality, this could be an explanation for the difference in performance of our model between centre 1 and centre 2.

To the best of our knowledge, only Yoo et al. [25] and Wang et al. [24] attempted a similar study using several ML strategies. Consistent with the results of both groups, we showed that an ML-based classifier can perform equal to, if not better than, physicians on certain metrics. A SPEC of 73% was found in the study by Yoo et al. [25] and 89% in the study by Wang et al. [24], while our study found a SPEC of 90%. Besides the SPEC, we also found an improved ACC compared to Yoo et al. [25] and Wang et al. [24] (88% vs. 81% vs. 86%). Our study found a highest NPV of 90%, which is higher than the value previously reported by Yoo et al. [25] (73–81%). A possible explanation for the improved performance in our study is that we included primary tumour data and that we used a more advanced boosted decision tree. Besides that, our study was performed in a different patient population, which could result in small differences in performance. The NPV found in our study is comparable with the results of a meta-analysis of endoscopic ultrasound-guided fine-needle aspiration (89–91%) [28]. Conceivably, the present or a similar algorithm could replace or supplement endobronchial ultrasound procedures in patients who have [^18^F] FDG-avid lymph nodes in locations that are difficult to reach.

There are some important potential drawbacks associated with our study. For example, we used pathological data as a gold standard, but in clinical practice, surgically resected lymph nodes are hard to match with their counterparts in medical imaging. This might have also introduced a bias, which affects the reliability of our classifiers, although our results are consistent with the results of other groups. Additionally, we made use of manual segmentation of the image data, which was performed by a single experienced physician. Although this might have introduced some bias and small delineation errors, we do not believe that our results would have significantly changed by performing the segmentation task by multiple physicians. In clinical practice, however, this process should be automated before ML classification models can be applied routinely. Besides that, we used image data from two different hospitals. Although both centres are large regional hospitals with a general patient population, it is unknown how the software would perform in different patient groups, in different hospitals, and with different PET-CT scanners. To reduce the effects of overfitting, we used a fivefold validation and a test set from a different hospital. Ideally, the robustness of our classifiers should be evaluated in a large prospective study. When using a pooled dataset for training the classifier, we expect that this will result in similar performance between the training and test set. This suggestion is an important issue for future research. High performance of similar ML models on diagnostic CT data classification seems to suggest that even higher classification results might be possible and, therefore, we up-sampled and co-registered the PET volumes to the CT data; however, the performance of our model decreased when adding CT data features. An explanation might be respiratory motion artefacts and incorrectly registered diagnostic CT image data with PET-CT data. Therefore, we only used features based on the PET primary tumour and PET lymph node data.

## Conclusion

This study showed that using an XGB-based algorithm could potentially improve the performance of the classification of lymph node metastasis of NSCLC from PET images. In agreement with previous studies, we found that a simple threshold technique was inferior, but the XGB-based classifier models outperformed the human observers in terms of both negative predictive value and specificity. The inclusion of the primary tumour data improved the performance of the model, while additional knowledge of distant metastases did not significantly improve the performance of our model.

## Data Availability

Not applicable.
